# Negative mood induction: Affective reactivity in recurrent, but not persistent depression

**DOI:** 10.1371/journal.pone.0208616

**Published:** 2019-01-15

**Authors:** Anne Guhn, Bruno Steinacher, Angela Merkl, Philipp Sterzer, Stephan Köhler

**Affiliations:** 1 Department of Psychiatry and Psychotherapy, Charité –Universitätsmedizin Berlin, Campus Mitte, Berlin, Germany; 2 Department of Psychiatry and Psychotherapy, Vivantes GmbH Wenckebach-Hospital, Berlin, Germany; 3 Fliedner Clinic Berlin, Outpatient Clinic for Psychiatry, Psychotherapy and Psychosomatics, Berlin, Germany; University of Queensland, AUSTRALIA

## Abstract

**Background:**

Despite the high clinical and epidemiological relevance of persistent depression, little is known about its specific psychopathology and whether it is distinct from recurrent depression. Depression in general has been associated with blunted affective reactivity but the evidence from previous studies is inconsistent. Here, we asked whether affective reactivity might differ between persistent and recurrent depression.

**Methods:**

Twenty patients with persistent depression, 20 patients with recurrent depression and 20 healthy controls (HC) were recruited. Both patient groups showed moderate symptom severity. All participants underwent a sad mood induction procedure. Affective reactivity was assessed with the Positive and Negative Affect Schedule (PANAS) before and after mood induction.

**Results:**

We found a striking difference in affective reactivity between patient groups. While the persistent group showed blunted reactivity to mood induction, the recurrent group demonstrated an affective response that was comparable to HC, with an increase in negative and a decrease in positive affect. Blunted affective reactivity was thus specifically associated with persistent in contrast to recurrent depression.

**Conclusions:**

These results highlight affective reactivity as an important psychopathological feature that differs between the two patient groups. Preserved affective reactivity to emotional stimuli in the recurrent group might reflect a resilience factor against persistence of depression.

## Introduction

One third of the individuals with depressive disorder develop a chronic course with depressive episodes that persist for at least two years [1]. Compared to patients suffering from single or recurrent depressive episodes, patients with persistent depression are associated with a higher rate of psychiatric comorbidities (e.g. [2])and a higher number of non-successful treatment attempts [3, 4]. Thus, there is a growing interest in differentiating between persistent and recurrent depression, as they may require different therapeutic strategies [5]. However, the evidence for psychopathological differences that could shed light on risk or resilience factors of depression persistence is scarce.

According to current clinical classification (DSM-5 [6]), the diagnosis of persistent depressive disorder (PDD; 300.4) refers to symptoms that persist for at least two years without remission of more than two months at a time. In this regard, PDD refers to one continuous depressive episode, whereas patients who suffer from recurrent depression, that is, multiple episodes separated by remissions, are diagnosed with major depressive disorder (MDD; 296.xx). MDD further includes four additional symptoms, i.e. diminished interest, psychomotor agitation or retardation, weight loss and suicidal ideation, which are not included in PDD. The critical diagnostic feature for both, persistent and recurrent depression, is depressed mood, which is characterized by decreased positive affect or increased negative affect or both [6]. Mood disturbances are thought to alter emotional responses to emotional stimuli, a concept that is called affective reactivity. Accordingly, empirical studies have shown that patients with depressive disorders in general exhibit reduced affective reactivity to both positively and negatively valenced emotional stimuli [7]. However, there is considerable heterogeneity in findings between studies, and attempts to identify moderators that reliably explain variation in affective reactivity have remained elusive.

One possible explanation is that the *severity of depression* moderates affective reactivity. It has been proposed that severe depression might be associated with blunted affective reactivity while patients with mild to moderate depression may even show increased emotional responses [7]. Accordingly, severely depressed patients showed blunted affective reactivity to negative pictures [8]; and patients who reported a history of MDD but were in remission at the time of study participation responded with increased negative affect to negative stimuli in comparison to a healthy comparison (HC) group without a depression history [9]. Alternatively, variation in affective reactivity might be moderated by the *persistence of depression*, i.e. persistent depression might be associated with blunted reactivity whereas patients with recurrent depression may show normal or even increased reactivity. Indeed, we recently found patients with persistent depression to be unaffected by a negative mood induction that comprised the combined presentation of sad music with sad pictures depicting other person’s suffering [10]. Blunted susceptibility to emotional stimuli corresponds to the etiological concept of persistent depression that presumes a disconnection from the social environment due to early childhood maltreatment [11]. In this regard, childhood trauma was found to be associated with persistence of depression [12, 13] as well as an unfavorable treatment outcome [14]. However, whether blunted affective reactivity to mood induction is specific to persistent depression or whether it is a feature of depressive mood irrespective of depression persistence is still unknown.

The present study was designed to answer the question whether patients with persistent and recurrent depression differ in affective reactivity. To this aim, we recruited two groups of patients, who were carefully characterized with respect to persistence including childhood maltreatment and matched for severity of depression, as well as a HC group. Persistence in this regard refers to the duration of symptoms, rather than recurrence of episodes. Thus, we explicitly recruited patients who suffered from at least the second major depressive episode, but had recovered in the meantime, since we assumed that intermittent recovery from depression could be a critical distinguishing feature that may shed light on resilience factors against persistence of depression. All participants were confronted with the same sad mood induction protocol. Since the existing literature did not provide a priori hypotheses on either positive or negative affective reactivity in persistent versus recurrent depression, we hypothesized that patients with persistent depression would show a stronger general reduction in affective reactivity to sad mood induction than patients with recurrent depression.

## Methods

### Participants and procedure

The institutional review board “Charité’s Ethics Committee” of the Charité –Universitätsmedizin Berlin approved the study. The capacity to consent was evaluated by ensuring that participants understood the purpose and procedure as well as potential risks of the study. The study sample consisted of three groups: 20 patients with persistent depression, 20 patients with recurrent depression and 20 HC. The results of two subsamples (15 patients with persistent depression versus 15 HC) have been published elsewhere [10]. At the time of study participation, patients were treated in inpatient or day clinic settings at three Departments of Psychiatry and Psychotherapy (Charité Campus Mitte, Vivantes Klinikum Wenckebach, Fliedner Klinik, Berlin). The majority of patients in the persistent group fulfilled the criteria for a concurrent MDD (n = 17), while due to inclusion criteria no one had a PDD diagnosis in the recurrent group. Thus, symptom severity is assumed to be well matched between groups. All patients received a stable antidepressant medication (Table 1). To be included, patients in the persistent group had to fulfill the criteria for a PDD according to DSM-5 [6]. Patients in the recurrent group had to fulfill the criteria for a recurrent MDD and were only included if they reported clear inter-episode remissions. Exclusion criteria were MDD with psychotic symptoms, schizophrenia, bipolar disorders, substance dependence with less than three months abstinence as well as organic psychiatric disorders. The HC group did not fulfill the diagnostic criteria for any current or past psychiatric diagnosis. Written informed consent was obtained from all participants and the local ethics committee approved the study. There was no financial reimbursement for study participation.

**Table 1:** Frequencies of psychiatric medication.

		persistent depression	recurrent depression	*p*
**antidepressants**	SSRI	5	9	.313
	SNRI	5	2	.405
	TZA	2	2	1
	MAO	3	2	1
	Other AD	5	8	.495
**antipsychotics**		3	5	.693
**mood stabilizer**		3	5	.693
**others**		1	4	.340
**no medication (n)**		0	1	1

Since the majority of patients took more than one substance, frequencies represent the number of patients taking a substance. SSRI included Escitalopram (n = 6), Sertraline (n = 4), Fluoxetine (n = 2), and Citalopram (n = 2). SNRI included Venlafaxine (n = 6) and Milnacipran (n = 1). TZA included Amitriptyline (n = 2), Nortriptyline (n = 1) and Doxepin (n = 1). MAO-inhibitors included Tranylcypromine (n = 5). Other AD included the antidepressants Mirtazapine (n = 7), Agomelatine (n = 2), Bupropion (n = 4) and Tianeptine (n = 1). Antipsychotics included Quetiapine (n = 7) and Aripiprazole (n = 1). Mood stabilizer include Lithium (n = 8). Others include Pregabaline (n = 5) and Zolpidem (n = 1). The significance value refers to Fisher’s exact test. For two patients (one in each group) data were not available.

All participants underwent a mood induction procedure that involved the presentation of sad pictures (IAPS [15] with mood-suggestive music (“Åses Tod” by Edvard Grieg) for 225 s. They were instructed that a series of pictures together with music would be presented and that each picture should be viewed attentively. The experimenter was seated in the same room to ensure that participants kept performing the task and looked at the screen attentively. For a detailed description please refer to Guhn et al. [10].

### Measures

All participants were screened for major psychiatric axis I disorders using the Mini-International Neuropsychiatric Interview (M.I.N.I. [16]). To clearly differentiate the two patient groups according to recurrence rather than persistence of depression, the patient group with recurrent depression visualized their course of illness on a life chart with the help of a trained psychotherapist. The life chart illustrated a time line on the x-axis and the severity of depressive symptoms from 0 to -10 on the y-axis. Euthymic mood represented the range from 0 to -2. Thereby, onset of depression, number of previous depressive episodes, inter-episode remissions and a duration of less than two years of the current episode were verified. The severity of depression was assessed by using the 21-items version of the Hamilton Depression Rating Scale (HAMD_21_ [17]. For study inclusion, patients had to have a sum score of at least 15 in the HAMD_21_. All participants further answered the Childhood Trauma Questionnaire (CTQ [18, 19], a self-report measuring early familial maltreatment regarding the domains emotional and physical neglect, emotional and physical abuse, as well as sexual abuse. Demographic and clinical characteristics were explored through a custom-made questionnaire.

Affective reactivity before and after mood induction was assessed with the German version of the Positive and Negative Affect Schedule (PANAS [20, 21]. This self-report measure is composed of 20 adjectives that indicate momentarily positive affect (PA), e.g. “active”, as well as negative affect (NA), e.g. “nervous”, on a 5-point scale ranging from very slightly or not at all (1) to extremely (5). The PANAS was assessed twice, before and after the mood induction procedure. Furthermore, cognitive reactivity was investigated by two short parallel forms of the Dysfunctional Attitude Scale [22], which were administered before and after the mood induction procedure, respectively. However, these results are not presented in the present paper.

At the end of the session, all participants were requested to evaluate the pictures concerning arousal and valence using a Likert Scale with a range from no arousal (1) to high arousal (9) and from very unpleasant (1) to very pleasant (9). Unfortunately, due to technical problems, a subset of the recorded picture evaluation data was lost (remaining samples include n_CD_ = 8, n_ED_ = 18, n_HC_ = 19).

### Data analysis

The statistical analysis was performed with SPSS version 23 (IBM SPSS Statistics, Munich, Germany). Clinical and demographic sample characteristics as well as picture evaluations were compared using χ^2^-tests, Fisher’s exact test for group differences regarding the frequency of antidepressant medication and one-way analyses of variance (ANOVA). For the picture evaluations, the different sample sizes were considered by calculating Levene tests, which proved the homogeneity of variances (*ps* > .477), so that two ANOVAs for arousal and valence were conducted.

For affective reactivity, a 2 x 2 x 3 ANOVA with repeated measurements was calculated with PANAS (positive, negative) and time (pre, post) as within-subject factors and group (persistent, recurrent, HC) as between-subject factor. Threefold interactions were disentangled by reducing the group factor from three to two levels, i.e. persistent versus recurrent depression, persistent versus HC, as well as recurrent versus HC. Significant interaction effects were further elucidated by post-hoc Student’s *t*-tests at a significance level of *p* < 0.05 (two-tailed) to analyze specific group differences. Therefore, Cohen’s *d* is provided as an estimation of effect size (according to [23]. Absolute values for *d* ≤ 0.3 indicate small, *d* between 0.4 and 0.7 indicate moderate, and *d* ≥ 0.8 indicate large effect sizes [24].

Since our hypothesis was based on the assumption that affective reactivity is unaffected by severity of depression, we calculated Spearman’s correlation coefficients for the relationship between symptom severity and affective reactivity. Therefore, change scores for PA and NA from pre to post mood induction were correlated with sum scores of the Hamilton Depression Rating Scale (HAMD_21_) for the combined patient groups (e.g. ΔNA = post_NA_−pre_NA_). Furthermore, an exploratory analysis was performed for the association between childhood maltreatment and affective reactivity (ΔPA, ΔNA).

Multiple comparisons were accounted for with Bonferroni corrections (*p*_*corr*_). Non-sphericity was considered by applying the Greenhouse-Geisser correction.

## Results

### Demographic and clinical characteristics

**Fig 1:**
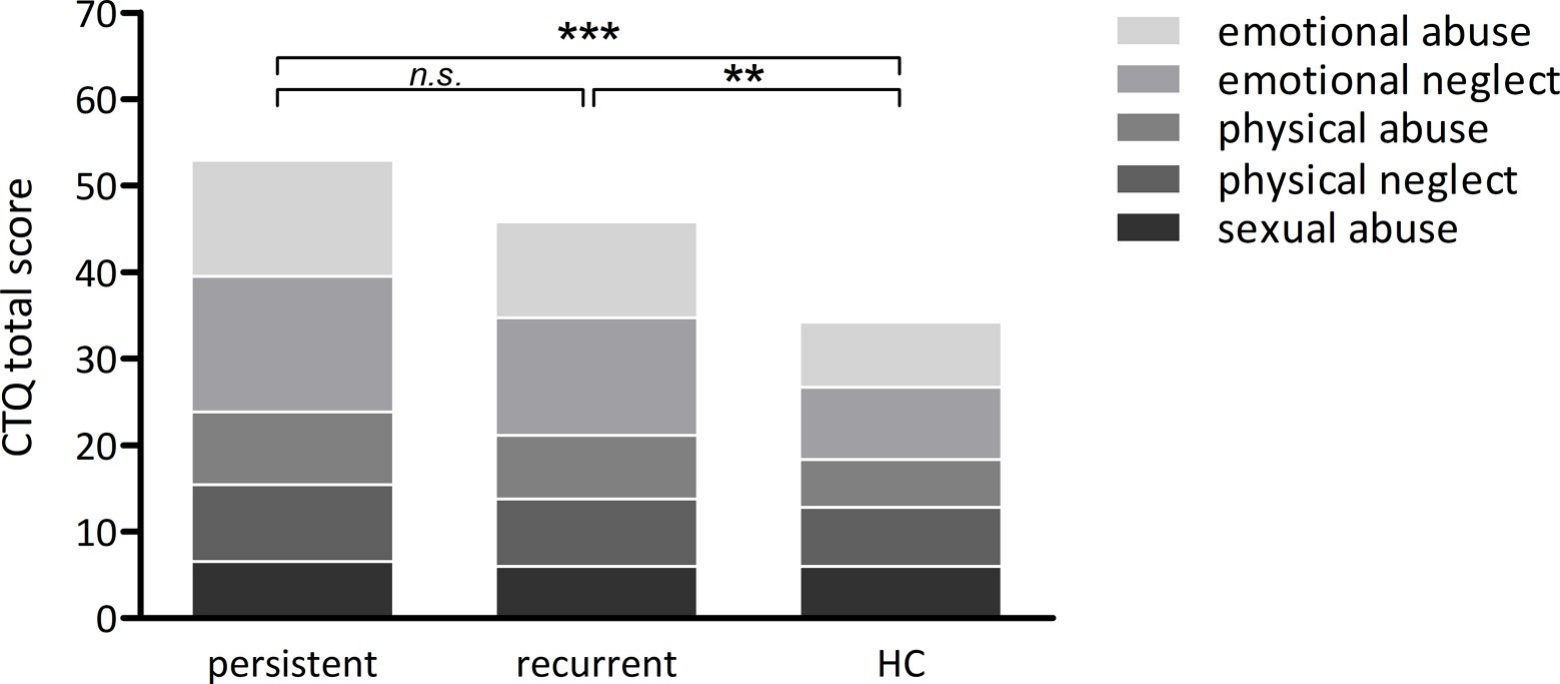
Group means on the amount of childhood traumatization for patients with persistent depression, recurrent depression, and a healthy comparison sample (HC). Asterisks indicate significant group differences concerning CTQ sum scores (*** *p* < .001, ** *p* < .01, *n*.*s*. non-significant).

Table 2 summarizes demographic and clinical characteristics of the study samples. Groups were well matched for age (F_(2, 59)_ = 0.55, *p* = .581) and gender (χ^2^_(2)_ = .549, *p* = .76). Patients of the persistent group were less likely to have current intimate relationship than the recurrent group and HC (χ^2^_(2)_ = 10.58, *p* = .005, *d* = 0.93). Both patient groups showed an equal level of currently present symptoms with a mean HAMD_21_ score of 20 corresponding to moderate severity (HAMD_21_: *t*_(29.3)_ = 1.3, *p* = .2, *d* = 0.4). There was further no group difference in age at first diagnosis (*t*_(35)_ = 0.36, *p* = .724, *d* = 0.1). There was no group difference in the amount of antidepressant medication, neither when comparing frequencies of single substances (*p* < .313) nor when comparing the number of patients taking medication at all (n = 19 CD, n = 18 ED, Table 1). The recurrent group showed a mean of 5.2 previous episodes (SD = 3.1, range: 2 to 15) with the current episode lasting for a mean duration of 16.2 weeks (SD = 12.8, range: 4 to 53 weeks). This sample thus suffered from a highly recurrent course of the disorder, which most likely reflects the inpatient setting in which they were recruited. As expected, groups differed with regard to childhood maltreatment [CTQ overall means(±SD): persistent group = 53(±17), recurrent group = 45.8(±15.5), HC = 33.6(±8), F_(6.5,184.6)_ = 4.57, *p* < .001] with a linear trend indicating that the amount of childhood maltreatment decreased from persistent to recurrent to HC group (F_(1,59)_ = 19.03, *p* < .001, Fig 1). Both patient groups showed a significantly higher amount of overall maltreatments than HC (*t*_(38)_ ≥ 3.1, *p* ≤ .009, *d* = 0.98), but did not differ statistically from each other (*t*_(38)_ = 1.39, *p* = .171, *d* = 0.4). One-way ANOVAs regarding the picture ratings revealed no group differences, neither for arousal (F_(2,47)_ = 0.16, *p* = .86) nor valence (F_(2,44)_ = 2.81, *p* = .072).

**Table 2:** Demographic and clinical characteristics of the study samples.

	persistent depression	recurrent depression	HC	*p*
**gender (m/f)**	9/11	7/13	9/11	.760
**mean age (SD)**	48.5 (10.1)	44.9 (11.2)	46.7 (11.3)	.581
**education**				
9 years	0	1	0	.053
10 years	9	13	5	
> 12 years	11	6	15	
**intimate relationship (yes/no)**	5/15	12/8	15/5	.005*
**age at depression diagnosis** (n = 3 missing answers)	33.7 (12.3)	32.4 (9.8)	-	.724
**HAMD_21_**	20.6 (2.4)	19.1 (4.5)	2.8 (3.3)	< .001*

Group differences for categorical variables were tested with non-parametric tests (χ^2^-test), for metric data the *p*-values of the *t*-statistic (age at diagnosis) and of the interaction term of a one-way ANOVA (age, HAMD_21_) are presented. HC = healthy controls, HAMD_21_ = Hamilton Depression Rating Scale (21 items version). Asterisks indicate statistically significant group differences (* *p* < .05).

### Affective reactivity

**Fig 2:**
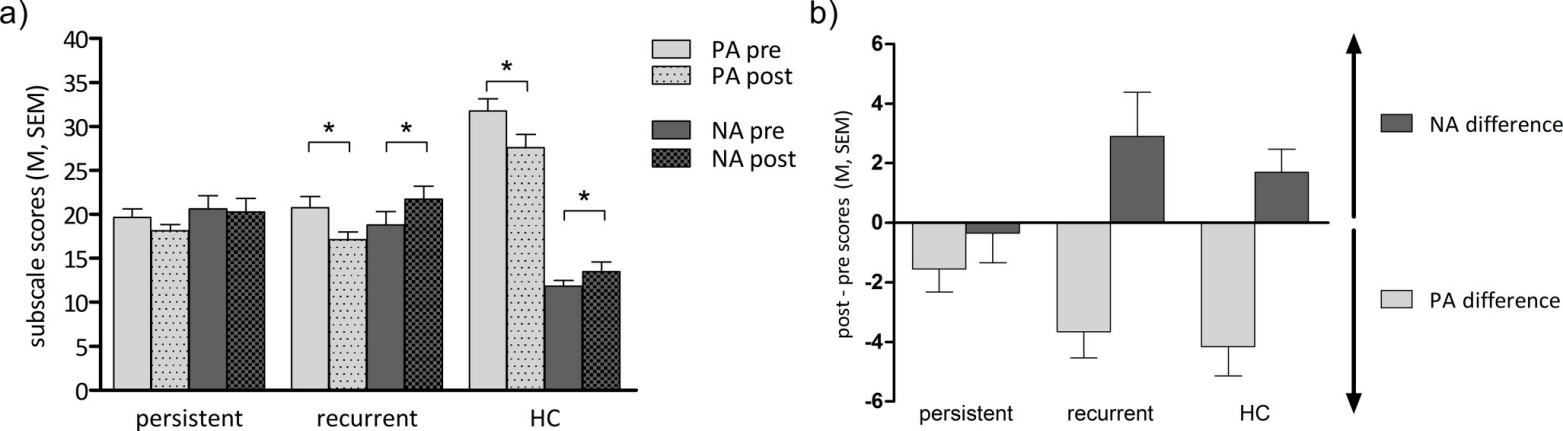
Affective reactivity. a) Group mean subscale scores with SEM for positive (PA) and negative affect (NA), pre and post mood induction, respectively. b) Difference scores (post-pre scores) for PA and NA per group. The greater the increase of NA as well as the decrease of PA, the higher the affective reactivity. persistent = patients with persistent depression, recurrent = patients with recurrent depression, HC = healthy comparisons. Asterisks indicate significant with-in group differences (* *p* < .05, Bonferroni corrected).

As we did not have separate a priori hypotheses for specific group differences in either positive or negative affective reactivity, we included both positive and negative affect in our statistical model. This resulted in a 2 x 2 x 3 ANOVA with the factors PANAS (positive, negative), time (pre, post) and group (persistent, recurrent, HC). We found main effects of PANAS (F_(1,57)_ = 22.52, *p* < .001) and time (F_(1,57)_ = 5.04, *p* = .029), showing an overall effect of mood induction on affect. There was a significant two-way interaction between the factors PANAS and group (F_(2,57)_ = 38.73, *p* < .001), indicating between-group differences in overall affect irrespective of mood induction. Both patient groups demonstrated a lower positive affect (PA) and a higher negative affect (NA) than the HC group (persistent vs. HC PA: *t*_(30.1)_ = -7.02, *d* = -2.2, NA: *t*_(30.4)_ = 4.79, *d* = 1.5; recurrent vs. HC: PA: *t*_(38)_ = -6.37, *d* = -2, NA: *t*_(38)_ = 4.87, *d* = 1.5, all *p* < .001; persistent vs. recurrent PA: *t*_(38)_ = -0.04 *p* = .969, *d* = -0.01, NA: *t*_(38)_ = 0.116, *p* = .908, *d* = 0.04). The ANOVA further revealed a significant two-way interaction between PANAS and time (F_(1,57)_ = 26.04, *p* < .001), that is, overall differential effects for positive and negative affective reactivity. Most importantly, there was also a significant three-way interaction between PANAS, time, and group (F_(2,57)_ = 3.57, *p* = .035), which indicates that the three groups were affected differently by the mood induction procedure (Fig 2). To disentangle the three-way interaction, three separate ANOVAs with only two group levels (persistent vs. recurrent, persistent vs. HC, recurrent vs. HC) were conducted.

The 2 x 2 x 2 ANOVA including both patient groups (persistent, recurrent) showed a significant PANAS x time (F_(1,38)_ = 12.49, *p* = .001) and a significant PANAS x time x group interaction (F_(1,38)_ = 5.95, *p* = .020). While there was no significant change over time for NA (*t*_(39)_ = -1.39, *p* = .172, *d* = -0.2), PA significantly decreased from pre to post mood induction (*t*_(39)_ = 4.3, *p* < .001, *d* = 0.6). However, this effect was only driven by the recurrent group (paired *t*-tests within recurrent group for PA: *t*_(19)_ = 4.15, *p*_*corr*_ = .004, *d* = 0.9; NA: *t*_(19)_ = -1.96, *p*_*corr*_ = .26, *d* = -0.4) while the persistent group did not show significant affective reactivity (PA: *t*_(19)_ = 2, *p*_*corr*_ ≥ .24, *d* = 0.4; NA: *t*_(19)_ = 0.35, *p*_*corr*_ > .73, *d* = 0.05).

The 2 x 2 x 2 ANOVA including persistent patients and HC as group factor levels revealed significant main effects for PANAS (F_(1,38)_ = 45.62, *p* < .001) and time (F_(1,38)_ = 66.51, *p* < .001) and significant interactions for PANAS x time (F_(1,38)_ = 14.85, *p* < .001) and PANAS x time x group (F_(1,38)_ = 6.46, *p* = .015). The persistent group reported lower overall PA (*t*_(30.14)_ = -7.02, *p* < .001, *d* = -2.2) and higher overall NA (*t*_(30.4)_ = 4.79, *p* < .001, *d* = 1.5) than the HC group. There was no change for NA (*t*_(39)_ = -1.06, *p* = .298, *d* = -0.1), but PA decreased significantly over time (*t*_(39)_ = 4.33, *p* < .001, *d* = 1.4). Again, this effect was specific for HC (PA: *t*_(19)_ = 4.15, *p*_*corr*_ = .004, *d* = 0.6; NA: *t*_(19)_ = -2.22, *p*_*corr*_ = .156, *d* = -0.4). The persistent group showed no significant changes for PA (*t*_(19)_ = 2, *p*_*corr*_ = .24, *d* = 0.4) nor NA (*t*_(19)_ = 0.35, *p*_*corr*_ > .73, *d* = 0.05).

The 2 x 2 x 2 ANOVA for recurrent patients and HC as group factor levels interestingly showed a non-significant threefold interaction between PANAS, time, and group (F_(1,38)_ = 0.081, *p* = .777), i.e. both groups were affected similarly by the mood induction procedure (PANAS x time: F_(1,38)_ = 25.431, *p* < .001). They both reported a decrease in PA (*t*_(39)_ = 5.92, *p*_*corr*_ ≤ .002, *d* = 1.9) and an increase in NA (*t*_(39)_ = -2.78, *p*_*corr*_ = .016, *d* = -0.9) after mood induction.

### Correlations

To test whether there was an impact of symptom severity on affective reactivity, Spearman’s correlation coefficients were calculated. Interestingly, for the combined patient group there was a trendwise negative correlation between HAMD_21_ score and negative affective reactivity (*r*_*s*_ = -.37, *p*_*corr*_ = .057, *d* = -0.8), i.e. a higher symptom severity was associated with a lower reactivity on negative affect. Within the persistent and recurrent groups, the correlation was non-significant (*p* ≤ .2). The re-analysis of the three-way ANOVA for the comparison between persistent and recurrent group on PANAS and time by controlling for HAMD_21_ as covariate (ANCOVA), however, did not change the above-mentioned results, i.e. the PANAS x time x group interaction remained significant (F_(1,37)_ = 4.46, *p* = .041). Moreover, there was neither a main effect of HAMD_21_ (F_(1,37)_ = 2.35, *p* = .13) nor a significant three-fold interaction with PANAS and time (F_(1,37)_ = 2.26, *p* = .14). For the HC group, there was no correlation between affective reactivity and the HAMD_21_ score (*p* ≥ .6).

**Fig 3:**
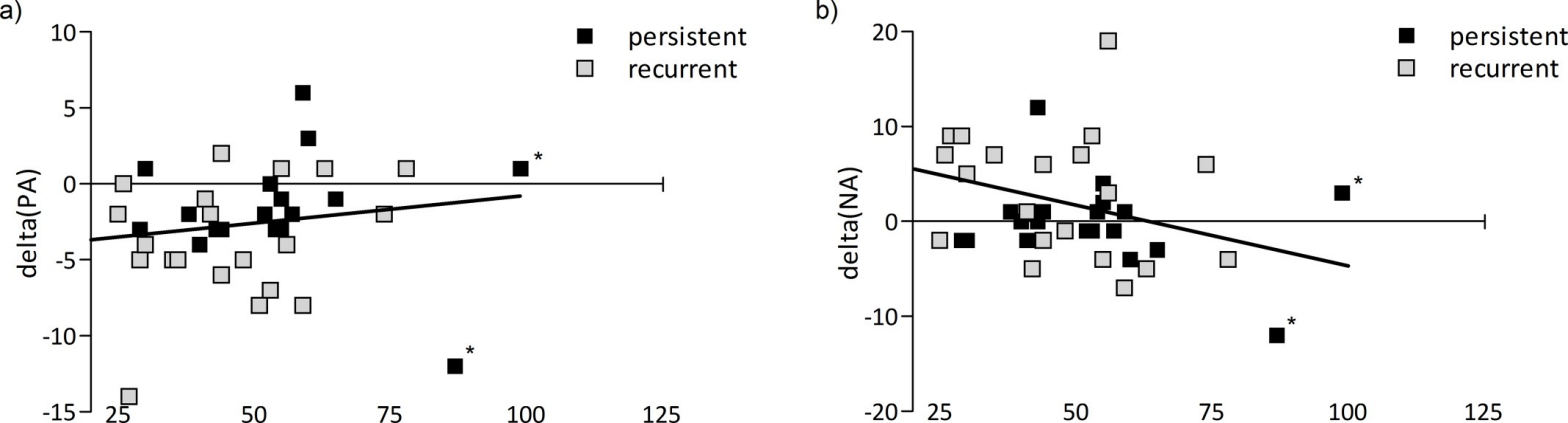
**Scatterplots for the relationships between changes in positive affect (ΔPA, a) and negative affect (ΔNA, b) on the y-axis with CTQ score (min = 25, max = 125) on the x-axis.** The analyses were performed on the combined group of persistent and recurrent patients, since CTQ scores did not differ between groups. For illustration purposes, the two groups are depicted in different colors. persistent = patients with persistent depression, recurrent = patients with recurrent depression. Asterisks denote outliers according to the interquartile-range method.

The exploratory correlation analyses with CTQ scores revealed two significant associations (Fig 3). First, childhood maltreatment correlated negatively with changes in negative affect (*r* = -.32, *p* = .041), and second, there was a trendwise significant positive correlation of childhood maltreatment with changes in positive affect (*r* = .28, *p* = .08). Please note that more negative difference values for positive affect reflect stronger reactivity (see Fig 2B) and that therefore the positive correlation with positive affect changes reflects more blunted affective reactivity in individuals with higher CTQ scores. Since patients with persistent and recurrent depression did not differ significantly in CTQ scores, they were poled for the purpose of these analyses. Using the interquartile-range method that considers data points outside the range between the first and the third quartiles as extreme values, we identified CTQ values from two patients as outliers. Exclusion of these patients even increased the correlation coefficient for positive affect change (ΔPA: *r* = .33, *p* = .043). The correlation for negative affect remained marginally significant (ΔNA: *r* = -.317, *p* = .053; n = 38).

## Discussion

The present study aimed at answering the question whether affective reactivity to mood induction in depression varies as a function of persistence of depression. Two groups of patients with persistent and recurrent depression were recruited who differed in terms of their course of illness, but were comparable with regard to the severity of depressive symptoms.

In accordance with our hypothesis, we found a striking difference in affective reactivity between persistent and recurrent patients: The persistent group showed blunted affective reactivity to mood induction, while the recurrent group demonstrated an affective reactivity that was comparable to the HC group, with increased negative affect and decreased positive affect. The present results thus support our hypothesis that persistence of depression is a key factor in determining affective reactivity to negative mood induction. This conclusion is further corroborated by three facts: (1) both groups suffered from an equal level of depressive symptoms, (2) both groups were on a similar amount of antidepressant medication, and (3) correction of statistical analyses with symptom severity did not change the results. However, the hypothesis that affective reactivity is moderated by symptom severity cannot be rejected, since negative affective reactivity was trendwise related to symptom severity in the combined group of persistent and recurrent patients. This is in line with earlier work demonstrating that diminished emotional reactivity is related to higher depression severity and psychosocial impairment [25, 26]. Thus, symptom severity might also have contributed to divergent results of previous studies [7]. However, the present findings contradict the meta-analytic finding of generally reduced emotionality in depression [7]. Rather, our findings suggest that blunted affective reactivity is a feature that differentiates persistent from recurrent depression.

Earlier work on mood induction in depression relied on cross-sectional data, e.g. HAMD scores, for study inclusion and largely neglected the previous course of the illness [27, 28]. It is therefore quite possible that divergent affective responses to mood induction will dissolve when patients with persistent and recurrent depression are mixed, or might yield inconsistent results between studies dependent on the degree of symptom persistence of the patient sample included. In our present study, the most relevant factor that distinguished both groups per inclusion criteria was the presence of inter-episode remissions from depression. The empirical evidence on epidemiological or psychopathological differences between persistent and recurrent depression that might explain differences in affective reactivity is scarce [29]. One replicated risk factor for depression persistence is childhood adversity [12, 14], especially in the form of emotional abuse and emotional neglect [13]. In a direct comparison between persistent and recurrent patients, some studies found persistent patients to score higher on self-reported early adversity than recurrent patients [30, 31] while others did not. Brakemeier et al. [32], for instance, found no difference in overall traumatization scores between the two patient groups, similar to the current data. In our sample, the persistent group reported a numerically higher degree of childhood maltreatment on all CTQ subscales except sexual abuse, but there was no significant difference between groups. Interestingly, exploratory analyses showed that childhood maltreatment across both patient groups was associated with reduced affective reactivity with regard to both positive and negative affect. This finding suggests that affective blunting may not only be related to persistence of depression but also to a history of childhood maltreatment. However, future studies with larger samples are needed to replicate these tentative findings and to answer the question whether childhood maltreatment may moderate the relationship between persistent depression and affective blunting.

Rottenberg and colleagues (2017), who found participants with a history of childhood-onset depression to report no affective response to a sad film clip, similar to our results, suggested that impaired empathy may be responsible for the blunted affectivity to sad mood induction [33]. Qiao-Tasserit and colleagues [34] provided experimental evidence for the association between empathy with others and affective reactivity. They found reduced behavioral responses to images of others’ pain after negative mood induction, particularly for those subjects who showed low empathy scores. However, the existing data on impaired empathy in persistent depression is rather inconsistent (e.g. [35, 36]). Patients with persistent depression were found to suffer from a higher impairment in social skills and higher levels of personal distress in social situations than patients with single or recurrent depression [37]. Thus, there may be a link between affective reactivity on the one hand and empathy and social skills on the other. Higher negative affective reactivity to an infant’s cry in a laboratory situation for instance predicted increased caregiving behavior [38]. On the basis of our findings, it is therefore tempting to speculate that intact affective reactivity as observed in the recurrent group may be a resilience factor against the persistence of depression. In other words, emotional susceptibility and presumably the social skills that are connected to this ability might facilitate prosocial behavior, which could in turn be a critical factor for recovery from depression. This speculation is on the one hand supported by the significantly higher frequency of intimate relationships in the recurrent group in the present study, and on the other hand, supported by previous findings that identified loneliness and social isolation as risk factors for recurrence and persistence of depression [39, 40]. Whether there is a connection between blunted affective reactivity and reduced empathy or social skills in depression persistence is an intriguing research question for future studies.

Interestingly, the recurrent group and the HC group showed a similar affective response to the stimuli. The finding of intact affective reactivity in the recurrent depression group is relevant for the interpretation of our findings in the persistent depression group. Taken alone, the absence of affective reactivity in the persistent depression group could reflect ceiling or floor effects, respectively, in the sense that high negative mood at baseline may not be further enhanced by sad mood induction and vice versa low positive mood may not be further reduced. However, the fact that patients with recurrent depression do show intact reactivity to mood induction, despite levels of negative and positive affect at baseline similar to patients with persistent depression, strongly argues against such ceiling or floor effects. The integration of this result into the existing literature, however, is difficult due to the insufficient characterization of patients with regard to depression persistence in former studies. Moreover, there might be a publication bias for significant group differences on affective reactivity, so that null findings are rather scarce. Sigmon et al. [27], for instance, did not find group differences between a HC group and current or remitted depressed patient group on the Depression Adjective Check List, which was assessed before and after presentation with audiotapes of positive and negative social scenes. While our results need to be replicated, they already point to affective reactivity as a critical factor with regard to the course of depression, which may also have important clinical implications. According to the distinction between mood and emotion (cf. [41], where mood is conceptualized as a slow-moving feeling state while emotions are quick-moving reactions to meaningful stimuli, the present results demonstrate that both patient groups suffer from mood disturbances, while patients with persistent depression additionally suffer from emotional disturbances. Thus, for patients with recurrent depression, therapeutic interventions that target mood disturbances, e.g. in the context of standard cognitive behavioral therapy, may be appropriate. Patients with persistent depression, in contrast, may require additional interventions that target the emotional disturbances reflected by altered affective reactivity. This assumption is supported by existing evidence showing that diminished affective reactivity predicts poor treatment outcome [25, 42]. The Cognitive Behavioral Analysis System of Psychotherapy (CBASP [11]) was specifically developed for the demands of patients with persistent depression. CBASP’s etiological concept is built on a disconnection between patients and their social environment due to early childhood maltreatment [11]. In favor of this hypothesis, the present results considered empirical evidence for blunted affectivity in response to stimuli showing the suffering of others. Mood induction with individualized autobiographical memories, in contrast, did induce affective and cognitive reactivity in patients with persistent depression [10]. Thus, there is no general impairment in mood reactivity. To overcome the interpersonal disconnection, which finds often expression in hostile and submissive behavior in these patients, CBASP uses the therapist-patient relationship for addressing perspective taking and empathy. In this regard, Constantino and colleagues [43] found that symptom improvement after CBASP was related to decreases in hostile-submissive behavior. Future studies are warranted addressing the hypothesis that a successful treatment specifically targeting blunted emotionality in persistent depression, e.g. with CBASP, likewise corresponds to increased affective reactivity to sad mood induction at the end of treatment.

To our knowledge, this is the first experimental evidence for differences in affective responses to negative mood induction between patients with persistent and recurrent depression. Although it cannot be excluded that some of the patients that were classified into the recurrent group may in the future maintain a depressive mood state that will eventually turn into persistent depression, we like to emphasize the careful investigation that was performed to recruit both patient groups. Regarding the existing literature, which is often lacking information on the course of illness prior to study inclusion, the characterization of both groups by onset, duration, course, severity of the depression as well as actual medication, this is considered a key strength of the present study. Of course, the strict distinction between the two groups in our study may appear somewhat artificial in the light of the natural heterogeneity of depressive disorders with respect to their course. However, we would like to argue that this consequent distinction was a prerequisite for identifying affective reactivity as a discriminative feature for persistent vs. recurrent depression, even in relatively small samples. This proof-of-concept study thus needs replication in larger samples.

In conclusion, the present study highlights affective reactivity as an important psychopathological feature that differs between persistent and recurrent depression. The present finding will aid the development and selection of effective strategies in the treatment of depression. Patients with persistent depression, for instance, might benefit from interventions that explicitly target affective reactivity, like CBASP, while such interventions may be ineffective in patients with inter-episode remissions. The finding of blunted affective reactivity as a specific feature of persistent depression raises many questions for future research. First, we need to find out whether blunted affectivity is a trait marker predicting persistence of depression or, the other way around, whether preserved affective reactivity predicts remission from depression. Alternatively, affective reactivity could become blunted in the first place if symptoms will not remit. Therefore, longitudinal designs are important that follow patients’ affective reactivity over time. Second, the correlation between affective reactivity and other psychological factors that maintain depression persistence needs to be investigated, since reduced empathy and interpersonal styles of hostility and submissiveness are likely related to blunted affectivity. In this regard, different emotional response systems such as psychophysiological, neural, or behavioral responses to mood induction may add important information. And third, from a clinical perspective, specific interventions that target emotionality in persistent depression should be examined for corresponding symptom improvement. Answers to these questions hold great promise for more individualized treatment strategies in depression.
