# Correction: Negative mood induction: Affective reactivity in recurrent, but not persistent depression

**DOI:** 10.1371/journal.pone.0213761

**Published:** 2019-03-07

**Authors:** Anne Guhn, Bruno Steinacher, Angela Merkl, Philipp Sterzer, Stephan Köhler

There is an error in affiliation 1 for authors Anne Guhn, Angela Merkl, Philipp Sterzer, and Stephan Köhler. The correct affiliation 1 is: Charité –Universitätsmedizin Berlin, corporate member of Freie Universität Berlin, Humboldt-Universität zu Berlin, and Berlin Institute of Health, Department of Psychiatry and Psychotherapy, Campus Mitte, Berlin, Germany.

The following information is missing from the Funding statement: The authors acknowledge support from the German Research Foundation (DFG) and the Open Access Publication Fund of Charité –Universitätsmedizin Berlin.
